# The advance of adjuvant treatment for triple-negative breast cancer

**DOI:** 10.20892/j.issn.2095-3941.2020.0752

**Published:** 2021-08-27

**Authors:** Jingyu Ge, Wenjia Zuo, Yiyu Chen, Zhiming Shao, Keda Yu

**Affiliations:** 1 Department of Breast Surgery, Fudan University Shanghai Cancer Center, Shanghai 200032, China; 2 Shanghai Medical College, Fudan University, Shanghai 200032, China

**Keywords:** Triple-negative breast cancer, adjuvant chemotherapy, targeted therapy, prognostic factors

## Abstract

Triple-negative breast cancer (TNBC) is a subtype of breast cancer characterized by its highly aggressive behavior, early recurrence, and poor outcomes, when compared with other subtypes. Due to the absence of the estrogen receptor, progesterone receptor, and human epidermal growth factor receptor 2 expression, TNBC lacks meaningful biomarkers and an effective therapeutic strategy. Chemotherapy remains the main adjuvant treatment for patients with TNBC. Anthracycline/taxane-based regimens are the standard of care in adjuvant settings. The addition of capecitabine or platinum may offer extra benefits to patients with TNBC, but at the cost of increased toxicity or adverse events. Dose-dense chemotherapy may enhance treatment efficacy in patients who are able to tolerate the treatment regimen, especially in high-risk patients. As a heterogenous disease, TNBC can be classified into several molecular subtypes according to genomic or transcriptional features, which may indicate potential targets for more precise and individualized treatment strategies. With our increased understanding of signal pathways associated with TNBC, as well as the discovery of novel biomarkers indicative of TNBC prognosis, several new therapeutic options are under investigation, and some have already reported good results. In this review, we summarized the current conventional therapeutic strategies and emerging clinical trials regarding adjuvant treatment for TNBC. Furthermore, we evaluated the prognostic value of several potential targets and the progress of targeted therapy in TNBC, both in neoadjuvant and adjuvant settings.

## Introduction

Breast cancers that lack expression of the estrogen receptor (ER), progesterone receptor (PR), and human epidermal growth factor receptor 2 (HER2) are categorized as triple-negative breast cancers (TNBCs), which comprise 10%–20% of all breast cancers. Clinically, TNBC patients tend to be younger than other subtypes and are more prevalent in African-American women, who have a higher possibility of harboring mutations in *BRCA1/2* breast cancer susceptibility genes^1^. Due to its high heterogeneity and aggressive behavior, TNBC patients have a higher risk of early recurrence and distant metastases, with higher likelihood of visceral metastases compared to bone metastases, leading to poorer outcomes^2^.

TNBC can be further divided into 4 or 6 molecular subtypes according to genomic or transcriptional features, which might indicate potential targets for a more precise and individualized treatment strategy. Due to its lack of meaningful therapeutic targets, chemotherapy remains the main systemic treatment for TNBC in neoadjuvant, adjuvant, and metastatic settings. This review will focus on the current literature and recent progress in the adjuvant treatment of TNBC patients.

## Adjuvant treatment for TNBC

TNBC lacks ER, PR, and HER2 expression and responds poorly towards endocrine and anti-HER2 therapies. Chemotherapy is therefore the preferred treatment for TNBC patients in neoadjuvant, adjuvant, and metastatic settings according to numerous clinical guidelines (e.g., the American Society of Clinical Oncology, the National Comprehensive Cancer Network, and the European Society for Medical Oncology). There is no evidence yet to indicate that a specific chemotherapy regimen is the most effective for TNBC. Anthracycline/ taxane-based regimens are therefore widely accepted as the standard treatment options. It is commonly acknowledged that anthracyclines can break DNA double strands, while taxanes can affect microtubule polymerization and depolymerization to disrupt microtubule dynamics and block mitosis, to inhibit the progression of tumors^3,4^.

Evidence from the CREATE-X study showed that the addition of adjuvant capecitabine in HER2-negative patients who did not achieve pathological complete response (pCR) after neoadjuvant chemotherapy could improve patient disease free survival (DFS) and overall survival (OS)^5^, but further research is needed to prove the benefit of additional capecitabine in TNBC patients receiving anthracycline/taxane-based adjuvant treatments. Due to the high prevalence of *BRCA1/2* mutations in TNBC patients, which often cause a deficiency in DNA repair pathways, the addition of platinum agents has been shown to lead to increased pCR percentages and improved outcomes^6,7^.

Several representative clinical trials of each regimen have been listed in **Table 1**^8–18^.

**Table 1:** Summary of trials of adjuvant treatments for triple-negative breast cancer

Adjuvant strategy	Study	Phase	Treatment	Primary endpoint
Anthracycline/taxane-based regimens	USO 9735^8,9^	III	AC*4 *vs*. TC*4	Disease-free survival
	ABC trial (USOR 06-090, NSABP B-46-I/OSOR 07132, and NSABP B-49)^10^	III	TC*6 *vs*. TaxAC	Invasive disease-free survival
	WGS Plan B^11^	III	EC*4-T*4 *vs*. TC*6	Disease-free survival
	GEICAM 9906^12^	III	FEC*4-wP*8 *vs*. FEC*6	Disease-free survival
	ECOG 1199^13^	III	AC*4-wP or wT or P every 3 weeks or T every 3 weeks	Disease-free survival
Capecitabine regimens	CALGB 49907^14^	III	Standard chemotherapy (CMF*6 or AC*4) *vs*. capecitabine	Relapse-free survival
	FinXX^15^	III	T*3-CEF*3 *vs*. TX*3-CEX*3	Relapse-free survival
	CBCSG-010^16^	III	T*3-CEF*3 *vs*. TX*3-CEX*3	Disease-free survival
	SYSUCC-001^17^	III	Standard treatment-X maintenance *vs*. standard treatment	Disease-free survival
Platinum regimens	PATTERN^18^	III	PCb*6 *vs*. CEF*3-T*3	Disease-free survival

A, doxorubicin; T, docetaxel; C, cyclophosphamide; TaxAC, one of several triple drug regimens that consist of cyclophosphamide, doxorubicin, and a taxane; E, epirubicin; F, fluorouracil; P, paclitaxel; wP, weekly paclitaxel; wT, weekly docetaxel; M, methotrexate; X, capecitabine; Cb, carboplatin.

### Traditional cyclophosphamide (CMF) and anthracycline/taxane-based regimens

The CMF (cyclophosphamide, methotrexate, and fluorouracil) regimens were the first combined chemotherapies to be used in adjuvant treatment of breast cancer patients, and have shown efficacy for the treatment of TNBC. The International Breast Cancer Study Group (IBCSG) VIII and IX trials compared patients who received 3 or 6 cycles of adjuvant classical CMF chemotherapy with or without endocrine therapy *vs*. endocrine therapy alone. Three immunohistochemically defined subtypes were included in this study: TNBC, HER2-positive and ER-absent, and ER-present. Although they found no clear chemotherapy benefit in ER-present disease [hazard ratio (HR): 0.90; 95% confidence interval (CI): 0.74–1.11], a significant benefit from chemotherapy was observed for the TNBC subtype, including a total of 303 patients (HR: 0.46; 95% CI: 0.29–0.73; interaction *P* = 0.009 between TNBC and ER-present disease)^19^.

A meta-analysis performed by the Early Breast Cancer Trialists’ Collaborative Group (EBCTCG) collated 123 randomized trials and evaluated them through log-rank breast cancer mortality rate ratios. After analyzing the long-term outcomes of 100,000 women, it concluded that patients treated with taxane-plus-anthracycline-based regimens or higher-cumulative-dosage anthracycline-based regimens had reduced breast cancer mortality at 10 years by approximately one-third, regardless of the breast cancer subtype. Furthermore, the study compared different polychemotherapy regimens for early breast cancer and found that a standard 4 cycles of doxorubicin plus cyclophosphamide regimen (AC*4) and standard CMF*6 regimens were equivalent in efficacy, with similar outcomes [rate ratio: 0.98; standard error (SE): 0.05; *P* = 0.67], but anthracycline-based regimens with substantially higher cumulative dosage (e.g., 6 cycles of fluorouracil, doxorubicin, and cyclophosphamide, FAC*6; or 6 cycles of fluorouracil, epirubicin, and cyclophosphamide, FEC*6) than standard AC*4 were superior to standard CMF*6 (rate ratio: 0.78; SE: 0.06; *P* = 0.0004)^20^.

However, long-term cardiotoxicity from anthracycline-containing regimens could not be ignored. Therefore, after the emergence of taxanes as an alternative option, several trials have evaluated whether other chemotherapy drugs could replace anthracycline. Unfortunately, different studies have reported inconsistent outcomes regarding the efficacy of anthracycline and taxane-based regimens. The USO 9735 trial compared AC*4 with 4 cycles of docetaxel plus cyclophosphamide (TC*4) in 1,016 operable patients with a median follow-up of 5.5 years. The TC*4 group presented with a superior DFS compared with the AC*4 group (86% *vs.* 80%; HR: 0.67; 95% CI: 0.50 to 0.94; *P* = 0.015). After a longer follow-up of 7 years, a significant difference in OS was found (87% for TC *vs*. 82% for AC; HR: 0.69; 95% CI: 0.50–0.97; *P* = 0.032)^8,9^. The ABC trials (USOR 06-090, NSABP B-46-I/OSOR 07132, and NSABP B-49) compared patients with HER2-negative breast cancer who received 6 cycles of TC (TC*6) or TaxAC regimens (AC regimen with a taxane). Their results showed that the 4-year invasive disease-free survival (IDFS) was 88.2% for TC*6 *vs.* 90.7% for TaxAC (*P* = 0.04). A significant difference was also observed in relapse-free survival (RFS), with 179 events occurring in the TC*6 group and 121 events in the TaxAC group (HR: 1.51; 95% CI: 1.20–1.90; *P* < 0.001). This trend was more obvious in hormone receptor-negative and node-positive patients, but there was no difference of the OS^10^. The WGS Plan B trial comparing EC*4-T*4 with TC*6 adjuvant chemotherapy reported similar 5-year DFS (89.6% *vs*. 89.9%) and OS (94.5% *vs*. 94.7%) outcomes in the 2 arms^11^.

Regardless of whether it may replace anthracycline, taxanes have been proven in numerous trials to be effective in the adjuvant treatment of breast cancer. The CALGB 9344 trial showed that the addition of sequential paclitaxel to standard AC*4 chemotherapy improved both the DFS and OS of early breast cancer patients with node-positive disease^21^. In this study, 3,121 women were randomly assigned to receive cyclophosphamide (600 mg/m²) plus doxorubicin (1 of 3 doses: 60, 75, or 90 mg/m²) for 4 cycles, followed by either no further therapy (AC*4) or 4 cycles of 175 mg/m² (AC*4-P*4) paclitaxel. The results showed that increasing a doxorubicin dose did not improve the outcome of these patients; for example, the 5-year DFSs were 69%, 66%, and 67% for patients who were randomly assigned to 60, 75, or 90 mg/m², respectively. However, the addition of paclitaxel improved the 5-year DFS by 5% (65% for AC*4 *vs*. 70% for AC*4-P*4) and OS by 3% (77% for AC*4 *vs*. 80% for AC*4-P*4). In an unplanned subset analysis, patients with negative ER status were shown to benefit more from the addition of paclitaxel (HR: 0.72; 95% CI: 0.59–0.86). In the GEICAM 9906 trial, 1,246 patients were treated with 4 cycles of FEC followed by 8 cycles of weekly paclitaxel (FEC*4-wP*8) or 6 cycles of FEC (FEC*6) alone. The 5-year DFS was significantly improved in the FEC*4-wP*8 arm (78.5% *vs*. 72.1%; *P* = 0.006). Furthermore, FEC*4-wP*8 treatment reduced the risk of relapse by 23% compared with the FEC*6 arm (HR: 0.77; 95% CI: 0.62–0.95; *P* = 0.022) and reduced the risk of death by 22% (HR: 0.78; 95% CI: 0.57–1.06; *P* = 0.110)^12^. In further studies of breast cancer subtypes, they found that patients with basal phenotypes benefited most from FEC*4-wP*8 treatment, with a significantly higher 7-year DFS (83% *vs*. 57%; *P* = 0.018)^22^. These studies showed that the addition of taxane may reduce the risk of recurrence and mortality, when compared to taxane-free chemotherapy, suggesting that taxanes played an important role in the adjuvant treatment of high-risk breast cancer patients, especially those with node-positive disease or TNBC.

The ECOG 1199 phase III trial evaluated the role of taxane and the schedule in operable breast cancer by comparing patients treated with 4 cycles of AC followed by paclitaxel or docetaxel every 3 weeks for 4 cycles, or weekly for 12 weeks, in which paclitaxel every 3 weeks was regarded as the standard arm. After a median follow-up of 12.1 years, a significant improvement in DFS was observed together with a slightly improved OS in the weekly paclitaxel (HR: 0.84; *P* = 0.011 and HR: 0.87; *P* = 0.09, respectively) and every 3 weeks docetaxel arms (HR: 0.79; *P* = 0.001 and HR: 0.86; *P* = 0.054, respectively). In the TNBC subgroup, weekly paclitaxel significantly improved both the patient DFS (HR: 0.69; *P* = 0.010) and OS (HR: 0.69; *P* = 0.019)^13^. The results suggested that 4 cycles of AC followed by weekly paclitaxel (AC*4-wP*12) may be the preferred choice for adjuvant chemotherapy of TNBC patients.

### Capecitabine regimens

As an oral prodrug of fluorouracil, capecitabine has shown high efficacy in the treatment of gastric cancer^23^, but its effectiveness in breast cancer remains controversial.

The CALGB 49907 trial compared standard chemotherapy (CMF*6 or AC*4) *vs*. capecitabine in breast cancer patients of 65 years or older, revealing standard chemotherapy to be superior to capecitabine, with RFSs of 56% and 50%, respectively (HR: 0.80; *P* = 0.03). Their 10-year update reported that RFS remained superior for standard adjuvant chemotherapy *vs*. capecitabine, especially in patients with hormone receptor-negative disease (HR: 0.66; *P* = 0.02)^14^. Based on these results, capecitabine is more commonly used in combination with other chemotherapeutic agents (such as anthracycline/taxane-based chemotherapy). Zhang et al.^24^ determined the efficiency and safety of pirarubicin plus capecitabine (PirX regimen) *vs*. pirarubicin plus cyclophosphamide (PirC regimen) adjuvant chemotherapy in 280 Chinese node-negative breast cancer patients. The 2 treatment arms showed similar 4-year DFSs (PirX *vs*. PirC, 93.6% *vs*. 92.9%; *P* = 0.761) and OS (PirX *vs*. PirC, 97.1% *vs*. 96.4%; *P* = 0.965)^24^, with less frequent severe toxicities and better health-related quality of life reported in the PirX regimen, suggesting that PirX may be a viable treatment option.

The FinXX study was the first to show that the addition of capecitabine to adjuvant chemotherapy regimens that contained docetaxel, epirubicin, and cyclophosphamide improved survival outcomes of TNBC patients^15^. This trial included 1,500 women from Finland and Sweden, with half of them (*n* = 747) receiving 3 cycles of docetaxel followed by 3 cycles of cyclophosphamide, epirubicin, and fluorouracil (T*3-CEF*3), while the remaining patients (*n* = 753) received 3 cycles of docetaxel plus capecitabine followed by 3 cycles of cyclophosphamide, epirubicin, and capecitabine (TX*3-CEX*3). The results showed that capecitabine-containing chemotherapy did not prolong the RFS (HR: 0.88; 95% CI: 0.71–1.08; *P* = 0.23) or OS (HR: 0.84; 95% CI: 0.66–1.07; *P* = 0.15) in the overall cohort, but RFS (HR: 0.53; 95% CI: 0.31–0.92; *P* = 0.02) and OS (HR: 0.55; 95% CI: 0.31–0.96; *P* = 0.03) benefits were observed in patients with TNBC.

A meta-analysis focusing on the role of capecitabine in the adjuvant and neoadjuvant therapy of breast cancer included 12 randomized clinical trials of early breast cancer, and the primary endpoint was the effect of capecitabine on DFS, including 2 subsets (1 added capecitabine to original regimen and the other replaced 1 drug of the original regimen with capecitabine). Their results showed that addition of capecitabin did not significantly improve the DFS of patients when compared to patients treated without capecitabine (HR: 0.95; 95% CI: 0.89–1.01; *P* = 0.115), but a significant improvement in OS was observed (HR: 0.89; 95% CI: 0.82–0.96; *P* = 0.005). In the TNBC subgroup (*n* = 3,854), capecitabine-containing regimens significantly improved the DFS (HR: 0.89; 95% CI: 0.79–0.94; *P* = 0.040) and OS (HR: 0.83; 95% CI: 0.72–0.95; *P* = 0.008) compared to regimens without capecitabine. However, this benefit was only observed with the addition of capecitabine (HR for DFS: 0.82; 95% CI: 0.71–0.94; *P* = 0.004; and HR for OS: 0.78; 95% CI: 0.66–0.92; *P* = 0.004) and not in replacement of another drug by capecitabine (HR for DFS: 1.07; 95% CI, 0.86–1.33, *P* = 0.531; HR for OS: 0.95; 95% CI: 0.74–1.21; *P* = 0.665)^25^.

The CBCSG-010 trial was further designed to validate the subgroup findings in the FinXX study. A total of 585 TNBC patients were randomly assigned to either control treatment (T*3+CEF*3) or capecitabine treatment (TX*3+CEX*3). After a median follow-up time of 67 months, the 5-year DFS was found to be significantly improved in the capecitabine group, when compared with the control treatment (86.3% *vs*. 80.4%; HR: 0.66; 95% CI: 0.44–0.99; *P* = 0.044), with similar improvements observed in the 5-year RFS (89.5% *vs*. 83.1%; HR: 0.59; 95% CI: 0.38–0.93; *P* = 0.02) and 5-year distant DFS (89.8% *vs*. 84.2%; HR: 0.63; 95% CI: 0.40–1.0; *P* = 0.048). However, there was no significant difference in 5-year OS percentages between the 2 groups (93.3% *vs*. 90.7%; HR: 0.67; 95% CI: 0.37–1.22; *P* = 0.19). Regarding adverse events, the most common toxicities were grade ≥ 3 hematological toxicities, including neutropenia (45.8% *vs*. 41.0%) and febrile neutropenia (16.8% *vs*. 16.0%). Patients who received capecitabine treatment also had a higher possibility of hand-foot syndrome (52.5% *vs*. 33.0%). Other adverse drug reaction such as alopecia, nausea and vomiting, peripheral neuropathy, and fatigue were similar between the 2 groups^16^.

Recently, the SYSUCC-001 trial, focusing on the benefits of metronomic capecitabine maintenance therapy, also reported encouraging results. In this study, 424 TNBC patients were randomized to receive capecitabine maintenance therapy (*n* = 222, 650 mg/m² bid continuously for 1 year) or observation (*n* = 221). A significant improvement in the 5-year DFS was observed in the capecitabine group (82.8% *vs*. 73.0%, HR: 0.64; 95% CI: 0.42–0.95; *P* = 0.03). Although no significant difference was observed in the 5-year OS (85.5% *vs*. 81.3%, HR: 0.75; 95% CI: 0.47–1.19; *P* = 0.22), a slightly higher OS rate was observed in the capecitabine group^17^.

The above results suggested that the addition of capecitabine to standard adjuvant chemotherapy may improve the prognosis of early-stage TNBC. However, capecitabine has not yet been recommended in clinical guidelines, and more clinical trials and further evidence are still needed.

### Platinum regimens

Studies have thus far shown that the addition of carboplatin to anthracycline/taxane-based chemotherapy regimens can significantly improve pCR in TNBC patients, but whether it will yield an improvement in clinical outcomes remains uncertain. In the GeparSixto study, patients were randomly assigned to receive paclitaxel (80 mg/m² once a week) and non-pegylated liposomal doxorubicin (20 mg/m² once a week) plus simultaneous bevacizumab (15 mg/kg intravenously every 3 weeks), with or without carboplatin^26^. The addition of carboplatin increased pCR percentages (53.2% *vs*. 36.9%; *P* = 0.005) but at the cost of hematological and non-hematological toxicities, including grade 3 or 4 neutropenia (65% *vs*. 27%), anemia (15% *vs*. < 1%), thrombocytopenia (14% *vs*. < 1%), and diarrhea (17% *vs*. 11%). These grade 3 or 4 hematological and non-hematological events significantly decreased (82% *vs*. 70%; 78% *vs*. 59%, respectively) when the dose of carboplatin was reduced from the area under curve by 2.0 to 1.5^6^. Further survival analysis revealed that TNBC patients treated with an addition of carboplatin had improved DFS (HR: 0.56; 95% CI; 0.34–0.93; *P* = 0.022), yet no statistically significant improvement in OS^27^.

Similarly, in the CALGB 40603 study, patients were treated with weekly paclitaxel, followed by doxorubicin plus cyclophosphamide every 2 weeks (wP*12-ddAC*4), and were then randomly assigned to receive concurrent bevacizumab with or without carboplatin. The results showed that addition of either carboplatin (60% *vs*. 44%; *P* = 0.0018) or bevacizumab (59% *vs*. 48%; *P* = 0.0089) significantly improved the pCR in breast cancer, but only carboplatin (54% *vs*. 41%, *P* = 0.0029) significantly increased the pCR in breast and axilla. Unfortunately, long-term RFS and OS outcomes were not recorded in this study^7^. More recently, Iwase et al.^28^ conducted a study regarding the outcomes of carboplatin when added as a part of neoadjuvant chemotherapy. In their study, 179 HER2-negative breast cancer patients were randomly assigned to receive PCb*4-CEF*4 (4, 3-week cycles of carboplatin and weekly paclitaxel followed by 4, 3-week cycles of CEF) or P*4-CEF*4 (4 cycles of weekly paclitaxel followed by 4 cycles of CEF). Their results showed that the addition of carboplatin to neoadjuvant chemotherapy significantly improved the DFS (HR: 0.22; 95% CI: 0.06–0.82; *P* = 0.015) and OS (HR: 0.12; 95% CI: 0.01–0.96; *P* = 0.046) in a subset of TNBC patients. Similarly, in a metastatic setting, the CBCSG 006 trial indicated that cisplatin plus gemcitabine may be a preferred chemotherapy choice for patients with metastatic TNBC^29^.

Finally, in an adjuvant setting, the PATTERN trial was the first to compare PCb*6 (6 cycles of paclitaxel plus carboplatin) with traditional CEF*3-T*3 (3 cycles of cyclophosphamide, epirubicin, and fluorouracil followed by 3 cycles of docetaxel) regimen. The trial showed that the carboplatin-containing arm had a significantly improved 5-year DFS (86.5% *vs*.80.3%; HR: 0.65; 95% CI: 0.44–0.96; *P* = 0.03), but there was no statistical difference of the OS (HR: 0.71; 95% CI: 0.42–1.22; *P* = 0.22). Nevertheless, this trial indicated that paclitaxel plus carboplatin may be an alternative adjuvant treatment choice for patients with operable TNBC^18^.

In conclusion, platinum regimens are promising therapeutic strategies for TNBC both in neoadjuvant and adjuvant chemotherapies, as well as in metastatic diseases. However, the adverse effect and toxicity of the drugs cannot be ignored.

### Dose-dense chemotherapy

Previous studies have revealed that dose-dense chemotherapy may enhance the efficacy of chemotherapy, especially in the high-risk breast cancer patients.

The WSG AM01 trial compared 236 high-risk breast cancer patients who were randomly treated with traditional dose-dense chemotherapy (4 cycles of E90C600 followed by 3 cycles of C600M40F600 every 2 weeks, EC*4-CMF*3) or high-dose chemotherapy (a rapidly cycled tandem high-dose regimen with 2 cycles of E90C600 every 2 weeks followed by 2 cycles of E90C3000Thiotepa400 every 3 weeks, EC*2-ECThiotepa*2) chemotherapy. After a median follow-up of 61.7 months, they discovered that both 5-year event-free-survival and OS percentages were highly improved in the high-dose arm [event-free-survival: 62% *vs*. 41% (HR: 0.60; 95% CI: 0.43–0.85; *P* = 0.004); OS: 76% *vs*. 61% (HR: 0.58; 95% CI: 0.39–0.87; *P* = 0.007)]. Further analysis showed that young women with TNBC benefitted the most from the high-dose regimen^30^.

However, results regarding the high-dose regimen in high-risk breast cancer patients are controversial. Some studies reported that a high-dose regimen had a significant effect on event-free-survival^31,32^ or OS^33^, while others claimed that there was no difference in prognoses between patients treated with dose-dense or high-dense chemotherapy^34^.

A phase III trial reported by Del Mastro et al.^35^ compared patients with node-positive early breast cancer who received either dose-dense chemotherapy (FEC-P or EC-P every 2 weeks) or standard-interval chemotherapy (FEC-P or EC-P every 3 weeks). After a median follow-up of 7.0 years, they found the 5-year DFS was 81% (95% CI: 79%–84%) in patients treated every 2 weeks and 76% (95% CI: 74%–79%) in those treated every 3 weeks (HR: 0.77; 95% CI: 0.65–0.92; *P* = 0.004), while the 5-year OS was 94% (95% CI: 93%–96%) and 89% (95% CI: 87%–91%), respectively (HR: 0.65; 95% CI: 0.51–0.84; *P* = 0.001). Although they did not observe any improvement in DFS outcomes with the addition of fluorouracil, they did show that dose-dense adjuvant chemotherapy improved the DFS, when compared to standard chemotherapy.

Recently, a meta-analysis from the EBCTCG combined data from 26 randomized trials comparing 2 weekly *vs*. standard 3 weekly chemotherapy schedules, which showed that patients treated with dose-intensive chemotherapy had fewer breast cancer recurrences than those treated with standard scheduled chemotherapy (10-year recurrence risk: 28.0% *vs*. 31.4%; rate ratio: 0.86; 95% CI: 0.82–0.89; *P* < 0.0001). In addition, the 10-year breast cancer specific mortality (18.9% *vs*. 21.3%; rate ratio: 0.87; 95% CI: 0.83–0.92; *P* < 0.0001) and overall mortality (22.1% *vs*. 24.8%; rate ratio: 0.87; 95% CI: 0.83–0.91; *P* < 0.0001) were also found to be reduced in patients receiving dose-intensive chemotherapy. Surprisingly, there was no significant difference between patients with ER-positive and those with ER-negative disease, which suggested that the reduction in recurrence with dose-intense chemotherapy may not have been related to ER status^36^.

Based on these discoveries, the National Comprehensive Cancer Network has listed dose-dense anthracycline/ taxane-based chemotherapy among the preferred treatments for HER2-negative tumors, an opinion which was also strongly supported by the St. Gallen early-stage breast cancer consensus. However, dose-dense chemotherapy needs to be used with caution, because the increase in dose-density was significantly correlated to more severe chemotherapy-related toxicity and adverse events. Close monitoring of side effects is therefore recommended during dose-dense chemotherapy.

## The molecular subtypes of TNBC

As a subtype with high heterogeneity, TNBC can be divided into several molecular subtypes that share similar genomic or transcriptional features and may have specific therapeutic strategies. By analyzing gene expression profiles from 21 breast cancer data sets and 587 TNBC cases, Lehmann et al.^37^ identified 6 distinct TNBC subtypes, including 2 basal-like (BL1 and BL2), an immunomodulatory (IM), a mesenchymal (M), a mesenchymal stem-like (MSL), and a luminal androgen receptor (LAR) subtype. BL1 and BL2 subtypes are characterized by their high expression of cell cycle and DNA damage response genes, as well as high Ki-67 mRNA expression, which suggested that these subtypes may be more sensitive to platinum agents. The IM subtype is comprised of immune antigens and genes involved in cytokine and core immune signal transduction pathways, containing high numbers of tumor infiltrating lymphocytes (TILs). The TILs play a strong prognostic role in early-stage TNBC, with studies suggesting that these patients may benefit from immunotherapy^38^. Both M and MSL subtypes are characterized by enriched expression of genes involved in the epithelial-mesenchymal-transition and growth factor pathways. Patients with these subtypes may respond to PI3K/mTOR inhibitors or Src/Abl inhibitors. LAR subtype is characterized by luminal gene expression and is driven by the androgen receptor (AR), indicating that patients with this subtype may benefit from anti-androgen therapy such as bicalutamide. In their additional studies, Lehmann et al.^39^ refined and revised their TNBC classification into 4 distinct subtypes (BL1, BL2, M, and LAR) after considering the transcripts of normal stromal and immune cells. However, Burstein et al.^40^ reported 4 distinct TNBC subtypes after analyzing the DNA and RNA of 198 TNBC tumors, including LAR, MES, basal-like immune-suppressed (BLIS), and basal-like immune-activated; each of which had diverse prognoses.

Similarly, another study conducted by Jiang et al.^41^ used multi-omics profiling to classify Chinese TNBC patients into 4 subtypes from a genomic and transcriptomics viewpoint. The 4 subtypes included LAR, IM, BLIS, and MES. The study found that *PIK3CA* mutations and copy number gains of chromosome 22q11 were more frequent in the Chinese cohort, when compared to a non-Asian cohort, and that the LAR subtype had more HER2 somatic mutations and *CDKN2A* (a crucial gene related to cell cycle regulation) loss. The study provided a comprehensive molecular profile of a Chinese TNBC cohort and broadened our understanding of TNBC^41^. Based on their subtype classification, Jiang et al.^42^ conducted the FUTURE trial to characterize the precision treatment of refractory metastatic TNBC. Their primary results showed the clinical benefit of subtyping-based targeted therapy.

Currently, TNBC subtyping is usually used for research purposes and has not yet been widely used in clinical practice. This is partially due to the complexity of genomic analysis and the high expense of genomic testing for TNBC patients, but this may change with future technological advancements and further development of individualized treatments.

## Potential targets and therapeutic strategies for TNBC

As a highly heterogeneous disease, TNBC should not be treated as a single disease entity, and individualized treatment should take into account the mutational landscape^43^ and clonal genotypes of each tumor^44^. With the advancement of sequencing technologies, the exploration of TNBC-related cellular pathways, and the discovery of meaningful prognostic factors, numerous clinical trials are being conducted in the search for an effective treatment target for TNBC.

On the basis of the subtype classification mentioned above, recent studies have focused on the characteristic pathways of each subtype and the discovery of targeted treatment or prognostic factors for each subtype (**Table 2**). For example, studies have determined whether the *BRCA* status and homologous recombination repair deficiency (HRD) guided the usage of platinum agents and poly ADP-ribose polymerase (PARP) inhibitors, and whether they could be prognostic markers for TNBC patients.

**Table 2:** Summary of potential pathways and prognostic factors in triple-negative breast cancer

Pathway/prognostic factor	% of TNBC with expression/mutation	Function	Prognostic significance	Drugs/therapies
*BRCA* mutation	10%–20% (germline mutation)	The repair of DNA double-strand break	*BRCA* mutation is related to higher DFS	PARP inhibitors
EGFR	20%–50%	Cell growth	High expression of EGFR is related to worse DFS and OS	Cetuxmab, panitumumab
VGFR	30%–60%	Cell proliferation and new vessel formation	High expression of VGFR is associated with shorter RFS	Bevacizumab
PI3K/AKT/mTOR	~25%	Cell growth and survival	Inhibition of this pathway may increase PFS	Capivasertib, temsirolimus, everolimus
AR	0%–53%	Cell proliferation	The prognostic value of AR is controversial	Bicalutamide, abiraterone, enzalutamide
PD-L1 protein	~20%	Downregulate T cell activation	PD-L1 positive patients can get higher PFS after treatment of immune checkpoint inhibitors	Pembrolizumab, atezolizumab

### *BRCA* status and HRD

The *BRCA1/2* tumor-suppressor genes encode proteins through homologous recombination that are essential for the repair of DNA double-stranded breaks. Cancers with *BRCA1/2* mutations are often unable to repair double-stranded breaks through homologous recombination, so these patients are likely to be more sensitive to DNA damaging compounds, such as platinum agents^6,7^, as well as to the PARP inhibitors. The prevalence of a germline *BRCA1/2* mutation was reported to be 11.2% in unselected TNBC patients, which is significantly higher than its prevalence in non-triple-negative breast cancers, and the *BRCA1* mutation was more common than *BRCA2*^45,46^. The HRD score was derived by combining 3 main characteristics, including numbers of telomeric allelic imbalances, large scale transition, and loss of heterozygosity, all of which will cause deficiency of the DNA repair system^47–49^. The SCAN-B trial used whole gene sequencing and HRDetect (an algorithm to assess the additional benefits of stratification based on whole gene sequencing) to evaluate the *BRCA* status or HRD in 254 TNBC patients. Their results showed that 59% of patients were classified as HRDetect-high and that these patients had higher percentages of IDFS (HR: 0.42; 95% CI: 0.2–0.87) and distant relapse-free intervals (HR: 0.31; 95% CI: 0.13–0.76) compared to HRDetect-low patients^50^. The study concluded that the level of HRD may be an independent prognostic factor for TNBCs, which may also be used to screen for patients who will benefit more from adjuvant chemotherapy.

The predictive value of *BRCA* in guiding the usage of platinum regimens currently remains controversial. In a metastatic setting, the TNT trial compared the efficiency of single agent carboplatin *vs*. docetaxel in advanced TNBC patients. Their results showed that for patients with germline-mutated *BRCA1/2* breast cancer, carboplatin had double the objective response rate (ORR) of docetaxel (68% *vs*. 33%; *P* = 0.01)^51^. However, in the abovementioned GeparSixto study, no significant increase was observed in the pCR by adding carboplatin to neoadjuvant chemotherapy in the subgroup of TNBC patients with *BRCA1/2* mutations [non-carboplatin arm: 6 of 24 (66.7%); carboplatin arm: 17 of 26 (65.4%)]. Notably, significant benefit from the addition of carboplatin was observed in patients without *BRCA1/2* mutations; 66 of 120 patients (55%) without *BRCA1/2* mutations achieved pCR in the carboplatin arm compared to 44 of 121 patients (36.4%) in the non-carboplatin arm [odds ratio (OR): 2.14; 95% CI: 1.28–3.58; *P* = 0.004]^26^.

### PARP inhibitors

PARP inhibitors are commonly used in patients with germline *BRCA1/2* mutations. PARP is an enzyme involved in base excision repair, a critical pathway during the repair of DNA single strand breaks^52^. The efficiency of PARP inhibitors in the treatment of breast cancer has been proven by 2 large phase III trials, OlympiAD and EMBRACA.

In the OlympiAD trial, patients with germline *BRCA1/2* mutations and HER-2 negative metastatic breast cancer were randomly assigned to receive either olaparib monotherapy (300 mg bid) or standard therapy selected by physicians (capecitabine, eribulin, or vinorelbine). The median progression-free survival (PFS) was significantly longer in the olaparib group than in the standard therapy group (7.0 months *vs*. 4.2 months; HR: 0.58; 95% CI: 0.43–0.80; *P* < 0.001). Notably, grade 3 or higher adverse events were also reduced in the olaparib group (36.6% *vs*. 50.5%)^53^. The EMBRACA trial focused on advanced breast cancer patients with a germline *BRCA1/2* mutation, in which 431 patients were randomly assigned to receive talazoparib or standard therapy. The median PFS was 3 months longer with talazoparib monotherapy than with standard therapy (8.6 months *vs*. 5.6 months; HR: 0.54; 95% CI: 0.41–0.71; *P* < 0.001) and the ORR was significantly higher in the talazoparib group (62.6% *vs*. 27.2%; OR: 5.0; 95% CI: 2.9–8.8; *P* < 0.001)^54^.

Based on these positive results in metastatic settings, several trials determined the role of PARP inhibitors in early stage TNBC, to test whether it led to an improved pCR or long-term clinical outcome. The I-SPY2 trial showed that the addition of veliparib in combination with carboplatin resulted in a higher pCR [51%; 95% Bayesian probability interval (PI), 36%–66%] than the control group (26%; 95% Bayesian PI, 9%–43%) in TNBC, but the toxicity was also more significant in the veliparib-carboplatin group^55^. In contrast, patients in the BrighTNess trial did not achieve a significantly higher pCR percentage when treated with paclitaxel plus carboplatin with or without veliparib (53% *vs*. 58%; *P* = 0.36), but the pCR was significantly increased, when compared with paclitaxel therapy alone (53% *vs*. 31%; *P* < 0.0001), indicating that carboplatin may have more influence on pCR than veliparib for TNBC patients^56^. Another study investigated the ability of a single agent oral talazoparib to achieve pCR and reported a residual cancer burden of 53% among patients with a known germline *BRCA* variant and operable breast cancer^57^.

### EGFR-targeted therapy

Epidermal growth factor receptor (EGFR) is a cell surface transmembrane tyrosine kinase receptor which is related to several significant pathways such as the MAPK, AKT, and JAK/STAT pathway. EGFR expression was seen in 20%–50% of TNBC patients and was reported to be a poor prognostic factor^58–60^. However, cetuximab, an anti-EGFR monoclonal antibody, failed to show high efficacy in the treatment of metastatic TNBC patients. The TBCRC 001 trial reported a response of 6% (2 of 31) in the cetuximab monotherapy group and a response of 16% (4 of 25) in patients treated with cetuximab plus carboplatin^61^. Two phase II trials by Nabholtz and co-workers showed that the addition of panitumumab or cetuximab to anthracycline/taxane-based neoadjuvant regimens only led to a slight pCR improvement (24%; 95% CI: 7.3%–40.7%) in operable TNBC^62,63^.

Due to these results, EGFR inhibitors have yet to be recommended as a therapeutic strategy for TNBC patients who overexpress EGFR.

### VEGF targeted therapy

Vascular endothelial growth factor (VEGF) is related to the proliferation of tumor cells as well as new vessel formation, playing an important role in breast cancer growth and metastasis^64^. A molecular subtype analysis showed that M and MSL subtypes were associated with higher signature scores for angiogenesis^65^. According to a study by Linderholm et al.^66^, VEGF levels were statistically higher in operable TNBC (median: 8.2 pg/μg DNA) than in non-TNBC (2.7 pg/μg DNA; *P* < 0.001), and these TNBC patients were observed to have a shorter RFS (HR: 1.8; *P* = 0.0023), breast cancer-specific survival (HR: 2.2; *P* = 0.004) and OS (HR: 1.8; *P* = 0.005) than patients in the non-TNBC group. Similarly, in another study, both the basal and triple-negative groups showed significantly higher microvessel densities (*P* = 0.017 and *P* < 0.001, respectively) than non-basal and non-triple-negative groups, and vascular invasion was associated with poorer survival outcomes^67^.

In adjuvant settings, 2 large trials evaluated the efficiency and safety of bevacizumab for the treatment of breast cancer. The result of the BEATRICE trial showed no significant improvement in OS with the addition of bevacizumab to chemotherapy, when compared with chemotherapy alone in early TNBC patients^68^. The E5103 trial also found that the addition of bevacizumab to sequential anthracycline and taxane-containing adjuvant therapy did not improve IDFS or OS for patients with high-risk HER2-negative breast cancer^69^.

In neoadjuvant settings, the GeparQuinto, ARTemis, and CALGB 40603 studies reached similar conclusions that the addition of neoadjuvant bevacizumab to standard chemotherapy increased the pCR but did not demonstrate a DFS or OS benefit^7,70,71^.

Overall, bevacizumab has shown limited effect in previous studies and is not regarded as a preferred strategy for TNBC treatment.

### PI3K/AKT/mTOR pathway targeted therapy

The PI3K signaling pathway is related to cell growth and survival, and the most relevant downstream nodes of this pathway are AKT and mTOR. The PI3K/AKT/mTOR pathway is thought to play a fundamental oncogenic role in cancers and is activated by the activating events in oncogenes *PIK3CA*, *AKT1/2*, and *MTOR*, or inactivating events in tumor suppressor genes such as *PTEN*, *INPP4B*, etc^72^. In TNBC, *PIK3CA* is the second most frequently mutated gene, ranked only next to *TP53*^65^, and mutations of this signal pathway occur in approximately 25% of primary TNBCs^72^, indicating that this pathway may be a potential therapeutic target in TNBC that could benefit many patients.

According to the molecular subtype classification by Bareche et al.^65^, the LAR and MSL subtypes are enriched in mutations of *PIK3CA* (55% and 23%, respectively) with significantly higher mutation percentages, when compared to other subtypes. Everolimus, an mTOR inhibitor, was shown to increase the PFS in patients with hormone-receptor-positive advanced breast cancer^73^, and several clinical trials also showed that the PI3K inhibitor, buparlisib, plus endocrine therapy was effective in patients with *PIK3CA* mutations^74,75^.

However, only a few studies regarding PI3K pathway inhibition in the adjuvant treatment of TNBC have been conducted. Buparlisib, a PI3K inhibitor, was tested in the BELLE-4 trial to evaluate whether its addition to paclitaxel would benefit HER2-negative patients. Surprisingly, the study found that patients in the TNBC group tended to have a shorter median PFS with buparlisib *vs*. placebo (5.5 months *vs*. 9.3 months; HR: 1.86; 95% CI: 0.91–3.79), and patients with *PIK3CA* or *PTEN* mutations, or loss of *PTEN* expression showed no improvement in PFS with the addition of buparlisib^76^.

The LOTUS trial investigated the efficacy of the AKT inhibitor, ipatasertib, in metastatic TNBC. The study found that patients who received ipatasertib plus paclitaxel achieved a longer PFS (6.2 months, 95% CI: 3.8 months–9.0 months) than those who were treated with placebo plus paclitaxel (4.9 months, 95% CI: 3.6 months–5.4 months), but higher rates of the grade 3 or worse adverse events such as diarrhea and neutropenia occurred in the ipatasertib group^77^. Similarly, the PAKT trial evaluated another AKT inhibitor, capivasertib, in metastatic TNBC. In this trial, 140 patients were randomized to receive either capivasertib plus paclitaxel or placebo plus paclitaxel. The results showed that both median PFS (5.9 months *vs*. 4.2 months; HR: 0.74; 95% CI: 0.50–1.08; *P* = 0.06) and OS (19.1 months *vs*. 12.6 months; HR: 0.61; 95% CI: 0.37–0.99; *P* = 0.04) were improved in the capivasertib group. Subgroup analysis from the PAKT trial revealed that patients with *PIK3CA*/*AKT1*/*PTEN*-altered tumors (*N* = 28) showed increased PFS benefit from capivasertib plus paclitaxel therapy, when compared to the placebo (9.3 months *vs*. 3.7 months; HR: 0.30; 95% CI: 0.11–0.79; *P* = 0.01)^78^.

A phase I study, Basho et al.^79^ assessed the efficacy of mTOR inhibitors (temsirolimus or everolimus) in combination with liposomal doxorubicin and bevacizumab in patients with advanced metaplastic TNBC (an identifiable surrogate of mesenchymal TNBC). Significant improvement of ORR was observed in patients with PI3K pathway aberrations, when compared with those without PI3K pathway aberrations (31% *vs*. 0%; *P* = 0.04), but there was no difference in terms of clinical benefit rate (44% *vs*. 45%; *P* > 0.99).

In summary, PI3K/AKT/mTOR pathways are promising targets in TNBC therapy, especially for those who carry mutated *PIK3CA*, *AKT* or *PTEN* genes. However, further study is needed to prove the efficacy and to determine the toxicity of these drugs.

### Anti-androgen therapy

AR is a member of the steroid nuclear receptor family, which is expressed in approximately 0%–53% of TNBCs^80^. However, the prognostic value of AR in TNBC is controversial. For example, Bhattarai et al.^81^ reported in their multi-institutional study that AR positivity may be a marker of good prognosis in American and Nigerian patient cohorts. However, it may be related to poorer outcomes in Norway, Ireland, and Indian patient cohorts, and it was neutral in the United Kingdom cohort.

Recent clinical trials have attempted to determine the role of AR in TNBC, using the AR inhibitors such as bicalutamide, abiraterone, and enzalutamide. A phase II trial of bicalutamide tested 424 patients with ER/PR-negative breast cancer for their AR status and found 12% of them to be AR-positive (immunohistochemistry > 10% nuclear staining). In the bicalutamide group, the 6-month clinical benefit was 19% (95% CI: 7%–39%), and the PFS was 12 weeks (95% CI: 11 weeks–22 weeks). No grade 4 or 5 treatment-related adverse event was observed in these patients^82^. Another phase II UCBG 12-1 trial evaluated the efficacy of abiraterone acetate plus prednisone in TNBC patients with AR positive (immunohistochemistry ≥ 10%) disease. Among the 30 patients who were eligible and evaluated for primary endpoints, the 6-month clinical benefit was 20.0% (95% CI: 7.7%–38.6%), including 1 complete response and 5 stable disease cases ≥ 6 months. The ORR was 6.7% (95% CI: 0.8%–22.1%) and the PFS was 2.8 months (95% CI: 1.7 months–5.4 months), respectively. The most common drug-related adverse events included fatigue, hypertension, hypokalemia, and nausea, but most were grade 1 or 2^83^. Enzalutamide, a potent second-generation anti-androgen drug, was shown to be superior to bicalutamide in 2 large phase II trials of prostate cancer^84,85^, and the MDV3100-11 study assessed the efficiency of enzalutamide by comparing enzalutamide alone or in combination with endocrine therapies in women with advanced breast cancer. The results showed that in patients with high expression of nuclear AR (immunohistochemistry ≥ 10%), 33% (95% CI: 23%–45%) achieved a 16-week clinical benefit, with a median PFS of 3.3 months (95% CI: 1.9 months–4.1 months), and a median OS of 17.6 months (95% CI: 11.6 months to not yet reached)^86^.

These trials suggested that anti-androgen therapy may be useful in TNBC patients with AR expression, but the mechanism of anti-androgen therapy remains unclear, and further study is needed to determine whether AR can be a useful biomarker or a therapeutic target in TNBC patients.

### Immunotherapy

Immunotherapy is an emerging and promising new strategy in cancer therapy, because the tumor microenvironment may have a strong influence on survival, invasion, and metastasis. According to multiple TNBC subtype classifications, the immune-related subtype accounted for 20%–30% in TNBC patients, indicating that immunotherapy may be an effective treatment^37,39–41^.

TILs are immune cells that infiltrate tumor tissues, including cytotoxic CD8^+^ lymphocytes, CD4^+^ T helper cells, T regulatory cells, and macrophages. Due to the high presence of TILs in TNBC, TILs are regarded as a prognostic biomarker in TNBC patients. In a subgroup analysis of patient treated with neoadjuvant therapy, increased TIL concentration was associated with a survival benefit in HER2-positive breast and TNBC patients, but was associated with worse survival in ER-positive/HER2-negative breast cancer patients^87^. Two large analyses confirmed the important role of stromal TILs in early stage TNBC^38,88^ and concluded that stromal TILs could potentially identify a subgroup of TNBC patients with an excellent outcome, who may be exempted from adjuvant chemotherapy^88^. In a neoadjuvant setting, the presence of tumor-associated lymphocytes was associated with a significantly increased pCR percentage (42% *vs*. 3%; *P* = 0.012) for patients undergoing anthracycline/taxane chemotherapy^89^, and the presence of TILs in residual disease after neoadjuvant chemotherapy was associated with a better outcome^90^.

Programmed cell death protein 1 is an immune check point receptor that can downregulate T cell activation after binding with its PD-L1 or PD-L2 ligand. PD-L1 is expressed in approximately 20% of TNBC patients, and the efficacy of anti-PD-L1 therapy has been shown by several clinical trials^91^. In the ongoing phase II I-SPY2 trial, patients treated with pembrolizumab plus neoadjuvant chemotherapy achieved a higher pCR percentage than the standard neoadjuvant chemotherapy control arm (60% *vs*. 22%)^92^. In the phase III KEYNOTE-522 trial, patients with early TNBC were randomly assigned to receive either pembrolizumab plus neoadjuvant chemotherapy (PCb followed by AC or EC) or placebo plus neoadjuvant chemotherapy (PCb followed by AC or EC). The results showed a higher pCR percentage in the pembrolizumab group compared to the placebo (64.8% *vs*. 51.2%, *P* < 0.001)^93^. In metastatic settings, the IMpassion130 trial equally assigned patients with untreated metastatic TNBC to receive atezolizumab plus nab-paclitaxel or placebo plus nab-paclitaxel. The results showed significant treatment benefit for the PD-L1-positive group, where the median PFS was 7.5 months *vs*. 5.0 months, respectively (HR: 0.62; 95% CI: 0.49–0.78; *P* < 0.001), and median OS was 25.0 months *vs*. 15.5 months, respectively (HR: 0.62; 95% CI: 0.45–0.86)^94^.

As a popular field of study in recent years, immunotherapy has provided benefits for TNBC patients, but currently lacks strong evidence in the adjuvant settings, which requires further validation before it may be incorporated into standard adjuvant treatment for TNBC patients.

## Conclusions

There is presently no standard adjuvant treatment regimen for TNBC patients, so chemotherapy is the main treatment. Anthracycline- and taxane-based regimens are the most widely accepted and routinely used in a clinical setting. The addition of capecitabine or platinum is still controversial, but they might be an alternative for some specific patients.

For clinicians, while suitable patients can be encouraged to participate in clinical trials testing the precision treatment of TNBC patients, current clinical guidelines and consensus are still the preferred choices for adjuvant therapy of most TNBC patients. Selected TNBC patients with cT1a or cT1b may be exempt from adjuvant chemotherapy, and alternative therapy such as traditional Chinese medicine following chemotherapy is also being studied for its possible benefits. For patients with early stage TNBC, standard chemotherapy followed by low dose capecitabine maintenance therapy may improve the DFS. The addition of carboplatin is likely to increase the pCR and may improve the DFS, but the potential side effects should also be considered. Evidence of targeted therapy based on subtype classification is still insufficient and needs more clinical trials.

As molecular features, signal pathways and mechanisms have been extensively studied in research and clinical trials, we expect that more effective biomarkers and therapeutic targets will be identified in the future for the adjuvant treatment of TNBC patients.
